# Treatment of de Garengeot’s hernia: a meta-analysis

**DOI:** 10.1007/s10029-018-1862-5

**Published:** 2018-12-07

**Authors:** S. Linder, G. Linder, C. Månsson

**Affiliations:** Department of Surgical Sciences, Uppsala University, 75185 Uppsala, Sweden

**Keywords:** Hernia, Appendicitis, Femoral, Garengeot

## Abstract

**Purpose:**

de Garengeot’s hernia is a rare entity in which the appendix is located within a femoral hernia and is almost invariably encountered incarcerated in an emergency setting with concomitant appendicitis. In the literature, there are mostly single-case reports. The purpose of the present study was to perform a review of the literature to study the incidence, pathogenesis, demographics, clinical presentation, laboratory and radiological investigations, differential diagnosis, delay in diagnosis and treatment, operative findings, surgical technique, histological findings, the postoperative course, use of antibiotics, and complications regarding de Garengeot’s hernia.

**Methods:**

A literature search was performed through PubMed with the following search terms, single or in combination: Garengeot, femoral hernia, and appendicitis. Additional references were also found within the articles, and two patients from Uppsala University Hospital were added.

**Results:**

Between 1981 and 2016, 70 publications were identified, and with the additional two patients, the present series comprised 90 patients There were 75 women (median age 73.0 years) and 15 men (median age 78.0 years). On examination, an inguinal mass was found in 87 patients (97%), which was painful and the cause of primary complaint in 67 patients (74%): the median duration of symptoms was 3 days. Radiological investigations or ultrasound were performed in 67 patients (74%); computed tomography was the most accurate with a positive diagnosis in 23/34 patients. Appendicitis was found in 76 patients, gangrenous in 23, and perforated in 9. The surgical approach was inguinal in 76 patients, including 15 with concomitant laparotomy. The preperitoneal route was chosen in six patients, and laparoscopy alone in four patients. A mesh/plug was used in 22 patients (7/22 normal appendix) and suture repair in 59 (4/59 normal appendix: *p* < 0.01). Complications were analysed in 79 patients and occurred in 11%. There was no mortality.

**Conclusions:**

de Garengeot’s hernia is rare, being indistinguishable from an incarcerated femoral hernia in general. A delay in surgery should be avoided but if needed, computed tomography may be used for differential diagnosis. Although there is no standard treatment, mesh material does not appear advisable in the presence of a perforation, and it is beneficial for the surgeons to perform their routine method rather than a specific technique.

## Introduction

Within inguinal hernia surgery, there are confusing eponyms. The most confusing are: Richter’s hernia, after August Richter [1], describes a hernia where only a part of the bowel’s circumference is incarcerated; Littre’s hernia, after Alexis de Littre, is an abdominal herniation of a Meckel’s diverticulum and can be inguinal, femoral, and umbilical [2]; Amyand’s hernia, after Claudius Amyand, describes the appendix within an inguinal hernia [3]. The condition where a femoral hernia contains the appendix was first described by Rene Jacques Croissant de Garengeot [4] and is now known as de Garengeot’s hernia.

The aim of the present study was to perform a review of the literature creating a large collected series of case reports including two new cases of de Garengeot’s hernias from Uppsala University Hospital. The study is focused on of the incidence, pathogenesis, demographics, clinical presentation, laboratory and radiological investigations, differential diagnosis, delay in diagnosis and treatment, operative findings, surgical technique, histological findings, the postoperative course, use of antibiotics, and complications in this rare condition. Thus, being the currently largest series of de Garengeot’s hernia valuable knowledge may be obtained.

## Case report 1

**Fig. 1:**
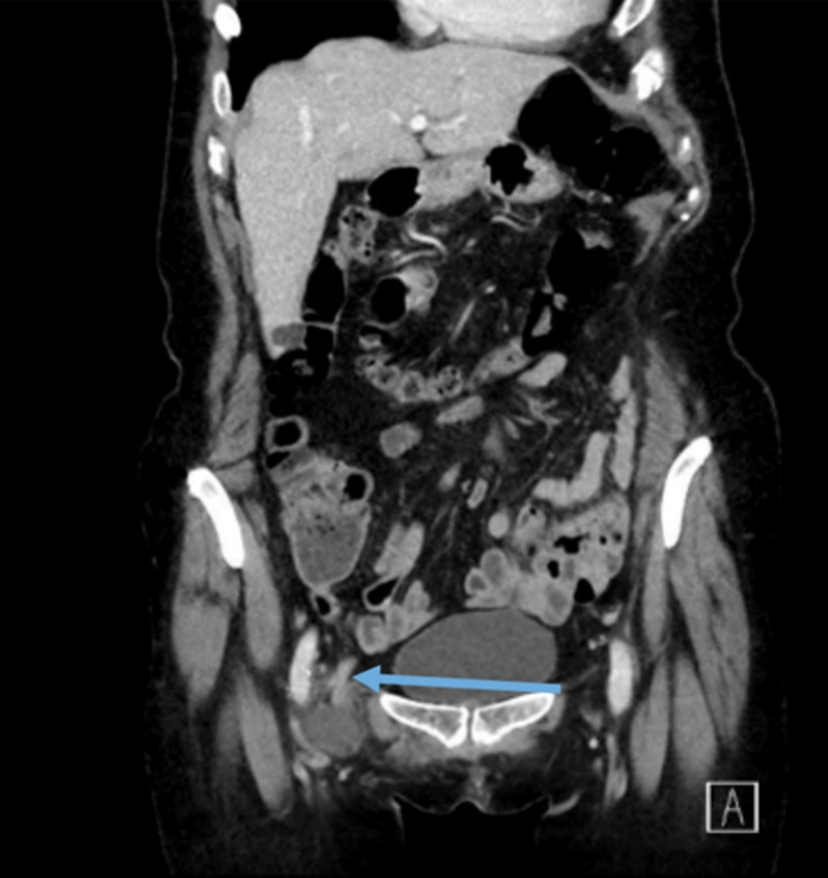
The Blue arrow shows the appendix going into the femoral hernia on the CT

**Fig. 2:**
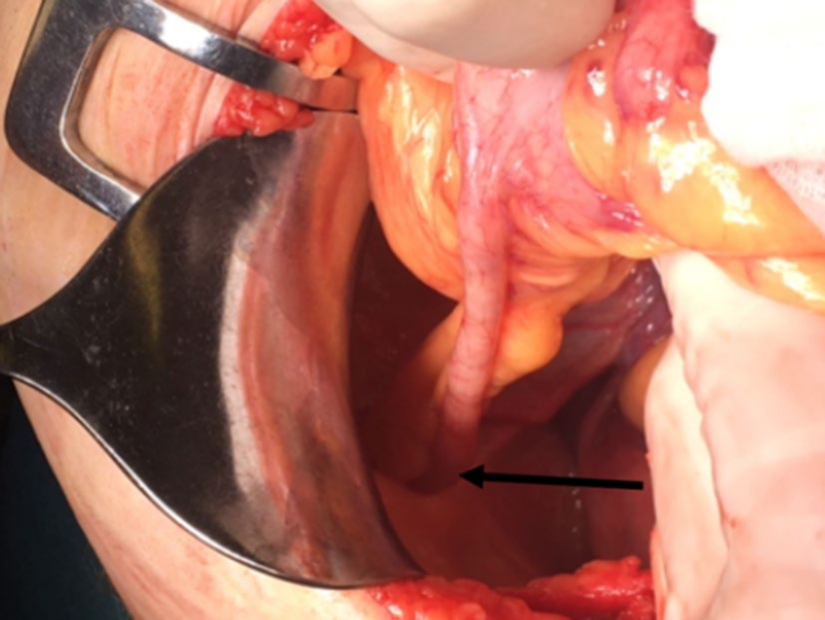
The black arrow shows the appendix going into the femoral hernia at the laparotomy

A 70-year old woman presented at the emergency department with a week’s history of painful swelling in the right groin. She had no symptoms of bowel obstruction and no fever. The CRP and white blood cell counts (WBC) were 1.1 mg/L and 5.2 × 10^9^/L, respectively (normal). Physical examination revealed a soft abdomen with painful swelling in the groin and a right-sided para-median incision, which she thought was due to some form of hernia operation, 25 years previously. The preliminary diagnosis was swollen lymph nodes, but an incarcerated femoral hernia could not be excluded. Computed tomography (CT) was performed and revealed a femoral hernia with an incarcerated appendix with fluid around the tip of the appendix (Fig. 1). The hernia could not be reduced and the patient went to surgery. A low midline incision confirmed the diagnosis of de Garengeot’s hernia (Fig. 2). As it was not possible to reduce the appendix from the hernia sac, a groin incision was performed. During the attempts to reduce the hernia, the appendix ruptured and it was extracted in pieces. Mesh repair was not chosen as the hernia was obviously contaminated; therefore, suture-repair was with prolene. The postoperative care was uneventful and the patient was discharged the next day. In the pathological examination of the appendix, there were signs of appendicitis but no malignancy. At the postoperative follow-up after 3 weeks, the patient was without any symptoms.

## Case report 2

A 73-year-old female smoker presented at the emergency department with a 2-day history of right-sided inguinal pain and difficulties in passing urine. She had a previous history of a pancreatic cancer and undergone pylorus-preserving pancreaticoduodenectomy in 2005. There was no fever and CRP and WBC were normal (1.8 mg/L and 4.4 × 10^9^/L). Upon examination, a 3-cm palpable aching mass in the right inguinal/femoral region was detected. There was no apparent erythema or other signs of infection in the cutaneous region overlying the mass. A CT revealed a suspected femoral hernia with adjacent inflammation and a tubular structure, presumed to be the vermiform appendix, in the hernia-sack. There were no radiological signs of small bowel obstruction. The patient received preoperative antibiotic prophylaxis (metronidazole, trimethoprim/sulfamethoxazole) and underwent open preperitoneal surgery with reposition of the hernia. The peritoneum was opened and an inflamed, but not perforated, appendix was found to be the content of the hernia. There were no signs of bowel obstruction at surgery. After appendectomy, a partially absorbable lightweight mesh (Ultrapro^®^) was placed and adhered with fibrin glue to cover the inguinal and femoral region. Postoperative clinical examination of the appendix revealed a transmural inflammation in the distal third of the appendix. No macroscopic tumour was present. The patient was discharged the day after surgery, but was readmitted 4 days after surgery due to constipation. The surgical-site seroma that developed was treated conservatively. All symptoms had resolved 4 weeks after surgery.

## Methods

To find studies on de Garengeot’s hernia published in the English-language, a literature search was performed through PubMed. The search terms used were Garengeot, femoral hernia, and appendicitis. The search term Garengeot only rendered patients published from 2005. By combining search terms and through references within the articles, further patients were found. Publications from 1981 to 2016 were included in the present series. Although certain individual data were difficult to extract in some series [5, 6], in the majority of the publications it was possible to evaluate relevant information. The two new patients from Uppsala University Hospital were also included and gave written and oral consent to be a part of the present series.

All publications included were scrutinized for information regarding incidence, pathogenesis, demographics, clinical presentation, laboratory and radiological investigations, differential diagnosis, delay in diagnosis and treatment, operative findings, surgical technique, histological findings, postoperative course, use of antibiotics, and complications.

In the diagnostic work-up, a plain abdominal film, ultrasound (US), CT or magnetic resonance imaging (MRT) were considered positive if a clear diagnosis of de Garengeot’s hernia was established, otherwise non-diagnostic (equivocal). Laboratory tests with WBC and CRP were specified as normal (within normal range), or elevated (any value above normal range). The surgical approach was classified as inguinal if the incision was in the inguinal region, below, at, or above the inguinal ligament (including a preperitoneal approach, McEvedy). A laparotomy was performed either as a midline incision or as an incision in the right lower quadrant.

## Statistics

Fisher’s exact test was used to calculate differences in the distribution of absolute numbers of patients. Student’s unpaired *t* test or Mann–Whitney *U* test were applied to analyse differences between groups of patients. Simple regression analysis was applied to analyse the duration of symptoms before operation and the length of hospital stay over time. All statistical analysis was performed with StatView^®^ version 5, SAS Institute Inc. (SAS Campus Drive, Cary, NC 27513, USA).

## Results

In the literature search, 70 publications comprising 88 patients were identified (Table 1). Thus, with the two new patients, this review included 71 studies covering 90 patients. Sixty-three publications comprised one patient each and three publications comprised two patients (including Uppsala university hospital). There were three and four patients in two series each and one study included seven patients. Some studied variables were not available in all case reports.

**Table 1:** From the literature search, 70 publications were identified comprising 88 cases of de Garengeot’s hernia

Ahmed et al. [7]	1 case
Akabri et al. [8]	3 cases
Akopian et al. [9]	1 case
Allen et al. [10]	1 case
Al-Subaie et al. [11]	1 case
Ardeleanu et al. [12]	1 case
Barbaros et al. [13]	1 case
Beysens et al. [14]	1 case
Brown et al. [15]	1 case
Caygill et al. [16]	1 case
Chung et al. [17]	1 case
Comman et al. [18]	1 case
Coskun et al. [19]	1 case
Couto et al. [20]	1 case
D`Ambrosio et al. [21]	1 case
Dholakia et al. [22]	1 case
Dulskas et al. [5]	4 cases
Ebisawa et al. [23]	1 case
Erdas et al. [24]	1 case
Filatov et al. [25]	1 case
Fitzgerald et al. [26]	1 case
Fukukura et al. [27]	1 case
Garcia-Amador et al. [28]	2 cases
Georgiou et al. [29]	1 case
Granvall et al. [30]	1 case
Guirguis et al. [31]	1 case
Halpenny et al. [32]	1 case
Hao et al. [33]	1 case
Hussain et al. [34]	1 case
Isaacs et al. [35]	1 case
Jin et al. [36]	2 cases
Jootun et al. [37]	1 case
Kevric et al. [38]	1 case
Khatib et al. [39]	1 case
Kokoszka et al. [40]	1 case
Konofaos et al. [41]	1 case
Le et al. [1]	1 case
Leite et al. [42]	1 case
Madiha et al. [43]	1 case
Maizlin et al. [44]	1 case
Mizumoto et al. [45]	1 case
Nguyen et al. [46]	1 case
Pan et al. [47]	1 case
Phillips et al. [48]	1 case
Piperos et al. [49]	1 case
Pitchaimuthu et al. [50]	1 case
Racy et al. [51]	1 case
Rajan et al. [52]	1 case
Ramsingh et al. [53]	1 case
Rebai et al. [54]	1 case
Rose et al. [55]	3 cases
Rossi et al. [56]	1 case
Salkade et al. [57]	1 case
Schäfer et al. [58]	1 case
Shah et al. [59]	1 case
Sharma et al. [6]	7 cases
Shum et al. [60]	1 case
Sibona et al. [61]	1 case
Sinraj et al. [62]	1 case
Suppiah et al. [63]	1 case
Talini et al. [64]	1 case
Tancredi et al. [65]	1 case
Tanrikulu et al. [66]	1 case
Thomas et al. [67]	4 cases
Thomas et al. [68]	1 case
Vos et al. [69]	1 case
Watkins et al. [70]	1 case
Whitehead-Clarke et al. [71]	1 case
Wiszniewski et al. [72]	1 case
Zissin et al. [73]	1 case

Two patients from Uppsala University Hospital were included in the present series

Among the cases, there were 75 women (83%) and 15 men (17%). The median age was 73.0 years (range 33–91) for women (*n* = 67) and 78.0 years (range 40–88) for men (*n* = 12). In 12 of the 90 patients (10 women and 2 men; 13.3%), there was documentation of a previous hernia in the inguinal region before the episode of the surgical procedure and a variation in time between some weeks up to several years.

On admission, the primary complaint was a painful inguinal mass (*n* = 67), abdominal pain (*n* = 10), a non-painful mass (*n* = 8), neither pain nor mass (*n* = 1), unknown (*n* = 4). An inguinal mass was documented in 87/90 patients. In one patient, the hernia was left-sided [16]. Fever was present in 14 patients but not found in 37, and information was lacking in 39. Erythema was found in 31 patients, but was not seen in 48, and was unknown in 11 patients. In patients with known information on fever, 9/20 patients with an erythema had fever as compared to 4/31 without erythema (*p* < 0.05). The median duration of symptoms was 3 days in both men and women (range 1 day, or less, up to a year). The duration of symptoms was not related to the presence of fever or erythema. A radiologically proven bowel obstruction was present in seven patients (8%), six women and one man. Laboratory tests, WBC and/or CRP were normal in 35 patients, elevated in 37, and unknown in 18 patients. There was no association between elevated laboratory tests and the presence of fever or erythema, and the laboratory test was not related to the duration of symptoms.

The diagnostic work-up included one radiological investigation or US in 52 patients, two investigations or more in 15, no radiology in 19, and no information regarding radiology in four patients. Abdominal plain films were obtained in 27 patients but none of the investigations was diagnostic of a de Garengeot’s hernia. A correct diagnosis was made on CT in 23/34 patients (68%), whereas one of the 18 US examinations could diagnose the condition (one was suggestive) (Table 2). Out of all patients, there was a delay in diagnosis and/or surgical treatment in nine patients Patient’s delay occurred in three patients, and a doctor’s delay in six, and the median delay was 4 days (1–120) (Table 3). The appendix was not perforated in any of these patients but gangrene was present in three patients, and the majority was discharged within 3 days.

**Table 2:** Radiological investigations and/or ultrasound used in the diagnostic work-up in patients with de Garengeot’s hernia (*n* = 90)

	Count
Single investigation	
No investigation	19
Abdominal plain films positive/negative	0/20
Ultrasound positive/negative	1/10^a^
CT positive/negative	13/8
Unknown	4
Multiple investigations	
Ultrasound negative and CT positive	5
Ultrasound negative and CT negative	1
Abdominal plain films negative and CT positive	4
Abdominal plain films negative and CT negative	2
Abdominal plain films negative and ultrasound negative	1
Magnetic resonance positive and CT positive	1
Magnetic resonance positive and ultrasound negative	1

Positive, diagnostic, and negative, non-diagnostic/equivocal findings

^a^In one patient, ultrasound was suggestive but not diagnostic

**Table 3:** There was a delay in diagnosis and/or surgical treatment in nine patients

Author	Delay (days)	Reason
Akopian et al.	7	Doctor’s delay. Infected lymph node? Antibiotics. Erythema disappeared. Planned exploration
Brown et al.	3	Doctor’s delay. Necrotic lymph node on ultrasound, scheduled for puncture after 7 days but returns earlier
Dholakia et al.	1	Doctor’s delay. Bowel obstruction clinically/X-ray, conservative treatment. Inguinal mass detected after 1 day
Madiha et al.	2	Doctor’s delay. Palpable inguinal mass with erythema, bowel content on ultrasound. Unclear delay
Mizumoto et al.	5	Patient’s delay. Palpable mass after coughing, progressively painful. CT diagnostic
Phillips et al.	7	Patient’s delay, avoided healthcare, ileus, inguinal hernia on CT. Appendix and perforated Meckel’s diverticulum found
Ramsingh et al.	14	Patient’s delay. Progressive increase in size, no pain. Appendix appeared normal, inflammation on histology
Tancredi et al.	3	Doctor’s delay. Reduction of hernia, recurrence 3 days later after colonoscopy
Watkins et al	120	Doctor’s delay. Drainage of inguinal abscess. A small mass explored electively, a fibrosed appendix was found

The most common surgical approach was inguinal; 58 patients (6 preperitoneal), and a combination of inguinal incision and laparotomy was chosen in 15 (Table 4). The femoral hernia repair was performed by suture techniques in 61 patients. A mesh was used in 22 patients of which two patients had a plug inserted. Some of the specified techniques were the McVay (*n* = 8), Cooper ligament repair (*n* = 8), and the Lichtenstein (*n* = 6). The choice of material used in the hernia repair according to the appearance of the appendix at operation is presented in Table 5. The use of a mesh was more frequent than a suture technique if the appendix was normal (*p* < 0.01). In the presence of a perforation, none of the authors used a mesh/plug. Drainage was used in nine patients in which six had a perforated or gangrenous appendix, and the skin was not primarily closed in four patients, all with a perforation or abscess.

**Table 4:** The surgical approach in the treatment of de Garengeot’s hernia (*n* = 90)

Surgical approach	Count
Inguinal^a^	58
Inguinal and laparotomy	15
Laparotomy	5
Laparoscopy and inguinal	3
Laparoscopy and laparotomy	1
TEP	1
TAPP	3
Unknown	4

*TEP* total extraperitoneal, *TAPP* transabdominal preperitoneal

^a^Inguinal repair, including six preperitoneal

**Table 5:** The choice of material used in the hernia repair according to the appearance of the appendix at operation (*n* = 81)

Material	Normal appendix	Appendicitis	Phlegmonous appendicitis	Gangrenous appendicitis	Perforated appendicitis
Suture, *n* = 59	4^a^	55^a^	31^c^	17^c^	8^c^
Absorbable^A^, *n* = 6	0^b^	6^b^	2	2	2
Non-absorbable^A^, *n* = 48	11^b^	37^b^	27	9	1
Mesh/plug^B^, *n* = 22	7^a^	15^a^	11^c^	4^c^	0

In seven patients, the type of suture material was not recorded. In addition, suture technique was used in one case with inconclusively described appendix and in one with chronic appendicitis. Some of the specified techniques used were: McVay (*n* = 8), Cooper ligament repair (*n* = 8), and Lichtenstein (*n* = 6)

^A^The type of suture material described, *n* = 54

^B^In two cases, a plug was used

^a^*p* < 0.01

^b^ns

^c^ns

At operation, an acute appendicitis was found in 76 patients (84%), among whom gangrene was present in 23 (30%) and a perforation in 9 (12%). In 12 patients, the appendix was normal. An abscess was encountered in six patients. In patients with a known duration of symptoms, the patient history before operation was longer in the presence of a normal appendix (median 14 days, *n* = 7) than if appendicitis was found in the hernia (median 3 days, *n* = 66) (*p* < 0.01). None of the patients with a normal appendix had fever (ns) or elevated blood tests (*p* < 0.01), and only two had an erythema (ns). In addition to the appendix, an incarcerated part of the small intestine was found in six patients (2 Meckel’s diverticulum), and in two patients, part of the caecum was within the hernia. Histology was obtained in 56/90 (62%) patients, confirming the diagnosis of a macroscopical appendicitis in all patients (one periappendicitis: Table 6).

**Table 6:** Distribution of the histological findings in relation to the surgical appearance

Surgical appearance	No histology	Appendicitis	Chronic appendicitis	Periappendicitis	Normal
Appendicitis, *n* = 76	27	48	0	1	0
Normal, *n* = 12	7	1	0	0	4
Chronic appendicitis, *n* = 1	0	0	1	0	0
Not described, *n* = 1	0	0	0	1	0

Histology was obtained in 56/90 (62%) of the patients

The presence of complications could be analysed in 79 patients and occurred in nine (11%). All had appendicitis, and in three, gangrene/perforation was found. A wound infection developed in four patients and a postoperative ileus in two patients. Other complications included wound dehiscence (*n* = 1), postoperative seroma (*n* = 1), serous wound leakage (*n* = 1), and reintubation (*n* = 1). Antibiotics were used in 38 patients, not administered to two patients, and information was lacking for 50 patients. The median postoperative hospital stay, documented in 59 (66%) patients, was 3 days (range 0–29 days). In the presence of a complication, hospital stay was prolonged, 3 days (range 0–21) to 11 days (range 1–29: *p* < 0.01).

## Discussion

This is a large series of patients with de Garengeot’s hernia collected from the literature, comprising 90 patients from 70 publications, including two from Uppsala University Hospital. There are two other large series, one with 31 reports and encompassing 36 patients [74], and the other analysing 50 articles with 64 patients [28]. Generally, the publications are case reports with only one patient. The paucity in patients with only scarce reports over a long period precludes firm conclusions on different aspects on the de Garengeot’s hernia, but a systematic evaluation of facts in a collected series may add valuable information.

## Incidence and pathogenesis

The true incidence of de Garengeot’s hernia is difficult to assess. It has been estimated there are between 100 and 200 patients in the literature [36, 74], but the exact figure is difficult to calculate. A frequency of 0.8%, accounting for 0.13% of all appendicitis, as reported in the literature, therefore, appears too high [36]. Thus, large register data are needed to assess the true incidence of de Garengeot’s hernia.

The entrance to the femoral canal, the femoral ring, is located posterior to the inguinal ligament, anterior to the pectineal ligament, lateral to the lacunar ligament, and is medial to the femoral vein. The femoral ring is narrow, fibrotic, and the limited space within the hernia increases the risk of incarceration [10, 36, 52]. Incarceration of the appendix in a femoral hernia may be promoted by a large caecum, a caecum positioned low in the pelvis, or an abnormal intestinal rotation [20, 65, 71]. Physical effort is reported to precede a de Garengeot’s hernia [64] and excessive weight loss and vaginal delivery or coughing also precede the incarceration [48]. The appendicitis is caused more by incarceration at the hernia neck than by appendicoliths [28].

## Demographics and clinical presentation

Femoral hernias constitute 2–4% of all groin hernia repairs [75]. In one series based on the Swedish Hernia Register [76], femoral hernias accounted for 2.8% of 141,916 patients: 67% of these were women, and among emergency femoral hernias, 74% were females. In a similar register study [77], the median age for emergency cases was 76 years, compared with 59 years for patients operated electively. In the present study, 83% were women, and the median age was 78.0 years for men and 73.0 years for women. Symptoms suggesting a known femoral hernia were only documented in 13.2%, for as long as up to 15 years [58, 62, 72]. This appeared to be less frequent than among common femoral hernias, in one large register study [78]; such findings were present in 202/433 (47%) emergency operations. In the present series only two patients were operated, on an elective basis [56].

Almost invariably, de Garengeot’s hernias are presented acutely when incarcerated and on clinical examination are indistinguishable from other incarcerated femoral hernias. In the present series, the most common clinical finding (87/90) was a mass in the inguinal region, of which pain was documented in 67. In comparison, the initial presentation of a lump in the groin was only 40% in a series of 406 emergency operations [79]. Diagnosis may be more difficult when patients present with abdominal pain alone (*n* = 10) [39, 59]. A complete abdominal examination includes the inguinal region, thus, avoiding that the condition is not diagnosed until CT is performed [1], or when the patient’s condition has deteriorated [22, 80].

Fever at presentation was documented in only 13 patients and was more frequent in the presence of an erythema (*p* < 0.05). In the present series erythema was a common finding (34%) and appeared to be more frequent than in the general setting. A probable explanation is that in many patients, fat is the content of a femoral hernia, and less inflammation is generated than if a part of the intestine is present. Formation of an abscess may occur [29, 44, 69]. One patient had a subcutaneous emphysema [55], and necrotising fasciitis has also been reported [31]. There was no obvious difference in duration of symptoms among patients with more serious complications than in the average patient in the series. With the narrow opening through the femoral ring, the inflammatory process is confined to a restricted compartment, thus promoting abscess formation. Therefore, peritonitis is uncommon and indicates a more serious condition, compared with the incarcerated femoral hernia in general [11, 52]. In a de Garengeot’s hernia it is not surprising that bowel obstruction is a rare event with an incarceration of the appendix alone and was found in seven patients [11, 52].

### Laboratory tests, radiological investigations, and differential diagnosis

Leucocytosis and/or elevated CRP was present in 37 patients but was not associated with longer duration of symptoms, fever, or erythema. It may be difficult to distinguish a femoral hernia from an inguinal hernia, not least in the presence of a large inflammatory process [10, 43]. Obviously, the location of the hernia in relation to the inguinal ligament helps to differentiate a femoral hernia (de Garengeot’s hernia) from an inguinal hernia (Aymand’s hernia), but a Richter hernia’s hernia also has similar features [67]. de Garengeot’s hernia may mimic lymphadenitis, lymphadenopathy, lymphoma, lipoma, abscess, or venous ectasia/thrombophlebitis [9, 74]. Abdominal radiographs were not diagnostic in any patient (*n* = 27) [12, 55, 72]. US was used in 18 patients, but the diagnosis was only established in one patient [25]. Conversely, US appears efficient in excluding a vascular component of the palpable finding, such as arterial aneurysm or thrombophlebitis [32]. A correct diagnosis was made in 68% of the 34 patients who underwent CT [19, 27, 28, 36–38, 61]. Differential diagnostic information is achieved, and the presence of bowel obstruction or abscess may be documented [15]. CT is a rapid investigation and is readily accessible; thus, surgery does not need to be delayed. However, with CT, the rarity of the de Garengeot’s hernia may increase the risk of misinterpretation and be underdiagnosed. The condition may instead be interpreted as an omentum [7], appendix not visualised [8], abscess formation [29, 68], bowel obstruction without bowel content in the femoral hernia [1, 48, 59], or small bowel in the hernia [69] (Table 2). MRT may be diagnostic and useful in the presence of an allergy to iodinated contrast [32] but is more time-consuming and less accessible than CT in many institutions.

### Delay in diagnosis and treatment

Doctor’s delay was the main cause of delay of diagnosis and treatment in six out of nine patients (Table 3). Two patients were evaluated as having lymph node enlargements [9, 15]. In one patient a bowel obstruction was treated conservatively, then a lump in the right groin was detected, and after CT the patient underwent surgery [22]. In three other patients with an inguinal mass, not detected on clinical examination, CT appears to have averted the delay [1, 59, 69]. Recurrence of the hernia after reduction delayed surgery in one patient, a 120-day delay occurred after drainage of an inguinal abscess, and in one patient the reason for delay was unclear [45, 48]. In three cases of patient’s delay, the symptoms were not alarming, thus postponing contact with healthcare [45, 48]. In patients with acute abdomen and suspected hernia through a palpable mass, a thorough examination of the inguinal region is essential to avoid delay [81]. In two series of patients with a fatal outcome after groin hernia surgery [82], there was a lack of initial groin examination in 41% and 37%, respectively, and also an association with imaging procedures causing a delay to surgery.

### Operative findings

Appendicitis was found in the majority of patients (84%), and in 42% of these patients, there was progression to gangrene or perforation: this is a larger proportion than for common appendicitis [83]. The finding of an abscess was associated with gangrene or perforation of the appendix [29, 44, 69]. There was no relation between the duration of symptoms and more advanced appendicitis. A common finding was the incarceration and inflammation of the distal part of the appendix, while the remaining, proximal part was unaffected [14, 33, 37, 61]: the entire appendix may also be within the hernia and inflamed [13, 68]. Preoperative diagnoses of subcutaneous emphysema and necrotising fasciitis were also confirmed at surgery [31, 55]. In 12 patients, a normal appearing appendix within the hernia was reported (17, 27); in these patients, blood tests were normal (*p* < 0.01), fever was absent, and erythema was only present in two patients. In order to reduce the risk of infectious complications an appendectomy was not performed on five of these patients: this is analogous with not performing incidental appendectomy in general and specifically the use of a mesh could be safer if no part of the bowel needs to be transected [6, 36].

The caecum was incarcerated along with the appendix in two patients: in one, reduction was possible after dilation of the hernia canal [36], and in the other, a right hemicolectomy was performed [21]. In four patients, a segment of the small bowel was found in the hernia; however, bowel resection was not necessary [1, 26, 39, 69], and in two patients with a concomitant Littre’s hernia, the bowel was resected [48, 51]. The finding of additional hernia contents indicates a larger entrance to the hernia; however, there was no previous history of hernia in any of these patients.

### Surgical technique

Generally, an inguinal approach alone was chosen, and provided access for both appendectomy and hernia repair (*n* = 58; Table 4 [20, 36, 56]). In most patients, incision was transverse, below [24, 40, 49], at the level of [41, 53], or above the inguinal ligament [45, 65, 66]. The preperitoneal route was only chosen in five previous patients [55]. This approach was also used in the second of the two patients from Uppsala University Hospital, and appendectomy could be easily performed by opening the peritoneum. In 15 patients, additional laparotomy was needed [24, 30, 48]. In emergency femoral hernia surgery in general, the need for laparotomy may depend on the surgical approach, and McEvedy’s technique (preperitoneal) appears to reduce the need for laparotomy more than Lockwood’s (infrainguinal) and Lotheissen’s (trans inguinal) approaches [84]. The main reasons for additional laparotomy in the present series were due to the base of the appendix being inaccessible through the inguinal approach and for facilitating abdominal exploration. These laparotomies were mainly as a McBurney incision [24] or a midline incision [30]. Five patients were managed by only laparotomy, by either a midline incision or mini laparotomy [10, 27]. Initial laparotomy was chosen if there was a suspicion of bowel incarceration [43], and in a patient with a preoperative diagnosis of an abscess with perforation [21].

In order to facilitate reduction of the hernia, some surgeons divided the inguinal ligament [23, 60] and the lacunar ligament [69]. The appendix is fragile, especially in the presence of gangrene or perforation, but a rupture of the appendix during operation, as in one patient from Uppsala University Hospital, appears to be a rare event. The appendix has also been divided to facilitate the procedure [68].

The inguinal approach was sometimes combined with laparoscopic interventions. In one patient, the base of the appendix was inaccessible and a laparoscopic appendectomy was successful [53]. Some authors chose laparoscopy as a diagnostic tool, and appendectomy was completed during the procedure [61]. In one report, the appendix was only transected at the base during laparoscopy and was subsequently removed inguinally [68]. In three patients, TAPP repair was completed after diagnostic laparoscopy [11, 18], and in another patient, TEP procedure was chosen after laparoscopic removal of the appendix [14]. Although Beysens et al. [14] highlight the risk of mesh contamination, they argue that with TEP repair, the mesh is placed outside of a possible contaminated area, thus minimising potential complications, as would be the case with open preperitoneal routes.

The most common hernia repair was through suture techniques (*n* = 61), and with non-absorbable material (Table 5). The two most frequently specified methods were the McVay [29, 58, 80], Cooper ligament suture techniques [46, 61, 65], and the Lichtenstein repair [50]. Many authors refrained from the use a mesh when there was severe inflammation, perforation, or in the presence of an abscess [21, 48, 59]. Twenty-two patients had a mesh repair, and in this group, the appendix was more frequently normal than in patients treated by a suture technique (Table 5). In order to reduce postoperative infection, a drain was inserted in nine patients [13, 29, 40, 55, 69], and the wound was not primarily closed in a further four patients [29, 55, 69]. These precautions were only taken in the presence of gangrenous appendix, a perforation or abscess, and were distributed over the whole study period. In comparison, suture repair dominated in emergency femoral hernia and constituted 930/1409 (66%) patients in a register study [77]. In the same series, mesh plugs and inguinal mesh were both used in 12%, open preperitoneal mesh in 8%, whereas the laparoscopic approach was only used in 24 patients [77].

There was wide variation in the choice of techniques in the present series. Surgeries on incarcerated hernias are emergency operations and thus not generally performed by “hernia experts”. New procedures develop, and thus the use of mesh material seemed to become more frequent during the study period, and the use of laparoscopically based procedures have emerged over time.

### Histological findings

A histological examination of the appendix was only performed in 62% of the patients (Table 6). Generally, the macroscopic findings were in agreement with the report on the surgical specimen from the pathologist. However, there is debate whether inflammation is a primary process in the appendix or if it is triggered by incarceration [18, 26, 34]. At an advanced stage with perforation or gangrene, this may be difficult to establish. Microscopical pathology may also be confined to the incarcerated portion of the appendix [1, 18, 67], Jin et al. [36] report that by releasing the neck of the hernia the perfusion normalised, and histology found no appendicitis. Fibrosis has also been found in specimens suggesting recurrent inflammations [70]. Furthermore, appendicolithiasis was rarely documented in specimens [39]. Thus, there is evidence supporting incarceration being the primary event with subsequent circulatory impairment and an inflammatory reaction, which may proceed to gangrene and perforation [31, 63]. Neoplastic changes in the appendix are not common, but in one patient, the histology revealed a villous adenoma [63].

### Postoperative course, use of antibiotics, and complications

In the present series, complication rate was (11%) including four post-operative infections (5%). In one large register study of emergency femoral hernia repair, complications occurred in 16.5% [77], and an infection rate up to 12% has previously been described [84]. A large percentage of common incarcerated femoral hernias contain only fatty tissue, and a bowel resection rate of some 23% is reported [77]. Thus, a higher infection rate could be expected in the present series, including patients with a gangrene/perforation, of which some also had a primary infectious complication. Antibiotics were used in all patients with post-operative infections [6, 7, 62]. One patient presenting with subcutaneous emphysema experienced uneventful recovery (skin left open) [55] but the post-operative course was not reported in a patient with necrotizing fasciitis [31].

Recovery was uneventful among patients with a diagnostic delay, and the majority was discharged within 3 days [9, 22, 45]. Hospital stay was prolonged in the presence of a complication and was similar to emergency hernia surgery in general [79]. In emergency femoral hernia a mortality up to 4–11% is reported [76, 77, 81, 85]. In the present series, there was no documented mortality. The follow-up time was generally short, and thus, no conclusions can be drawn regarding recurrent hernias.

## Conclusions

The acute clinical condition with the suspicion of an incarcerated hernia in the inguinal region often obviates the need for radiological investigations [71]. In some specific situations with differential diagnostic problems, CT appears the method of choice. de Garengeot’s hernia is rare, and operative treatment will not differ from that of a femoral hernia containing omentum or some part of the intestine, except for an appendectomy in most cases. The paucity of cases precludes a standardised operative procedure for de Garengeot’s hernia, and various methods are used in the present series. In the presence of a perforation, a suture repair may be chosen rather than a mesh to reduce the risk of a mesh-bound infection. Individual surgeons probably benefit from applying their standard techniques rather than choosing a specific method.
